# Newborn Genomic Sequencing Needs Confirmation but Not Repeating

**DOI:** 10.3390/children11111287

**Published:** 2024-10-25

**Authors:** Bruce Bennetts, Gladys Ho, Sarah Shin, Pak Leng Cheong, Tiffany Wotton, Enzo Ranieri, Shelley Pirreca

**Affiliations:** 1 Sydney Genome Diagnostics, Western Sydney Genetics Program, Sydney Children’s Hospitals Network, Westmead, NSW 2145, Australia; bruce.bennetts@health.nsw.gov.au (B.B.); gladys.ho@health.nsw.gov.au (G.H.); sarah.shin@health.nsw.gov.au (S.S.); 2 Specialty of Genomic Medicine, The Children’s Hospital at Westmead Clinical School, Faculty of Medicine and Health, University of Sydney, Westmead, NSW 2145, Australia; 3 Department of Medical Genomics, Royal Prince Alfred Hospital, Camperdown, NSW 2050, Australia; pak.cheong@health.nsw.gov.au; 4 NSW Newborn Screening Programme, The Children’s Hospital at Westmead, Westmead, NSW 2145, Australia; tiffany.wotton@health.nsw.gov.au (T.W.); enzo.ranieri@health.nsw.gov.au (E.R.); 5 Faculty of Health and Medical Sciences, The University of Adelaide, Adelaide, SA 5005, Australia; 6 Australian Genomics, Parkville, VIC 3052, Australia

**Keywords:** NBS, repeat testing, diagnostic

## Abstract

Newborn screening (NBS) has been one of the big innovations in public health. It has grown over the decades, especially with the introduction of tandem mass spectrometry. However, it is likely to expand significantly in the coming decades with the introduction of genomic testing. Traditionally, in NBS, there has been a pattern of repeat testing for confirmation and follow-up diagnostic testing. This follow-up is critical as NBS is a screening program. This pathway is appropriate for low-cost tests, but if public health authorities are going to invest in high-cost screening such as whole-genome sequencing, they are likely to baulk at repeating these expensive tests in a diagnostic setting. Our study investigates whether screening-grade data from NBS can be transitioned into diagnostic-grade data using a panel of single-nucleotide variants (SNVs) on a diagnostic specimen. These SNVs could be used to link the diagnostic specimen with all of the provenance requirements associated with routine pathology and the NBS genomic data. This strategy has large cost benefits and opens up the rapid use of NBS genomic data should a child present in an acute care setting and a genetic diagnosis is suspected. This approach will greatly speed up the confirmation of positive NBS results and reduce family anxiety due to delayed diagnostic testing.

## 1. A Brief Technical History of Newborn Bloodspot Screening

Newborn Bloodspot Screening (NBS) has been one of the most effective preventative public health developments in the last 60 years. It began with phenylketonuria in the 1960s [1] and has now expanded to a number of rare congenital disorders [2]. Screening tests were initially based on the measurement of metabolites as a discrete test. This was significantly enhanced by the adoption of tandem mass spectrometry (MS/MS) in the mid-1990s, resulting in the concept of a profiling test [3]. Over the last 50 years, NBS using dried blood spot (DBS) samples has evolved into the most contemporary technology of the time [4]. However, more recently, DNA-based technology has been introduced in NBS, initially as a second-tier test for disorders such as cystic fibrosis, introduced in 1989 [5], and additional variant analysis for inborn errors of metabolism (IEM) such as medium-chain acyl-coenzyme A dehydrogenase deficiency (MCADD) [6]. 

As stated by D Bailey ‘NBS (is) at a critical juncture in its evolutionary history, essentially a victim of its own success. NBS is the only viable mechanism to ensure universal identification of newborns who need early treatment for rare health conditions’ [7]. Globally as the world moves towards precision medicine, an equally global spotlight is shining brightly on NBS programs and the potentially expanded benefits these programs may offer in the future. James Bonham, MSc, PhD, CSci, FRCPath, President of the International Society for Neonatal Screening (ISNS), stated at the 2023 APHL/ISNS Newborn Screening Symposium in Sacramento that currently, nearly 50 million babies are screened globally on an annual basis, yet that is only about 28% of infants born every year [8].

## 2. Technological Advances

Technological advances in DNA methods have resulted in a number of groups adopting this as a first-tier assay where no measurable metabolite is available as a biomarker [9]. An example of this is the introduction of NBS for spinal muscular atrophy (SMA) and primary immunodeficiencies, such as severe combined immunodeficiency (SCID) [10,11]. The advantage of using a DNA-based assays is that they allow for the identification of individuals at increased risk of disease much earlier and prior to the development of symptoms. The success of NBS has opened the door to screening for a much larger group of disorders for which treatments or therapies are available. With over 700 genes associated with rare genetic disorders, for many, there are therapeutic approaches available, which has forged the concept referred to as the ‘Treatabolome’ (https://treatabolome.org, accessed on 17 August 2024). There is a recognition that with the advent of large next-generation sequence capture panels or whole-genome sequencing (WGS), this will be the future.

## 3. Screening Is Not a Diagnostic Test

NBS is considered screening and not diagnostic testing, and thus requires a diagnostic confirmation of any abnormal screening result. An NBS laboratory for all ‘positive screen’ results will request an additional DBS card collection for repeat testing, confirmation and, if warranted, the collection of appropriate diagnostic samples, which include urine and blood. Approved collection centres (ACCs) for diagnostic pathology collection purposes operate under strict governance frameworks of accreditation, policy and compliance, including specimen tracking with unique pathology IDs to accompany the specimen. There are many reasons why the original NBS result may require confirmatory testing. These include metabolite variability due to factors such as time of collection; environmental factors such as humidity, specimen contamination and collection errors; treatment effects; and maternal factors, with the clinical notes on the DBS card being relevant to the mother rather than the screening patient. 

## 4. NBS Paradigm Shift

The next paradigm shift in NBS is upon us, as comprehensive genomic sequencing in NBS is now being explored across the world [12]. The key questions are as follows: When and how might this method be implemented? Should we employ it? And can we afford it? By contrast, consider a scenario on which repeat sequencing (exome or genome) was not required, and was instead replaced by simpler versions of genomic testing such as variant-specific assays using PCR and Sanger sequencing performed as confirmation. These still have considerable costs and, more importantly, take time to design and perform. Our project on genomic testing in newborn screening funded by the Australian Medical Research Future Fund, entitled “Newborn Genomic Sequencing in screening: Therapy Ready and Information for Life (TRAIL)”, is exploring whether the NBS process can be simplified and made more cost-effective, whilst enhancing the reliability of the result. The TRAIL team will investigate the performance of a rapid panel of well-established single-nucleotide polymorphisms (SNPs) on a newly collected diagnostic specimen. The team will then link these specimens to data derived from genomic sequencing of NBS cards, which could be WGS, WES or a panel of genes which would include the relevant SNPs. This would establish that the two specimens, one derived from the NBS card and the other from a diagnostic collection, are in fact from the same individual.

The value of this approach is that it allows rapid confirmation of a newborn’s genomic information without the need to repeat a specific assay for the variants being investigated or repeating the whole genomic testing process. In fact, this approach provides greater validity to the provenance of the data and result compared to repeat testing on a second specimen, as the second specimen may have variability and faults associated with it. The method proposed by the TRAIL team not only ensures that the genomic result belongs to the child, but also proposes the data gained from screening can also be diagnostic data. The speed at which this can be achieved will allow for rapid diagnostic confirmation of the positive NBS result, reducing the delay in confirmatory diagnostic testing, which can often take weeks to months to complete. This will have significant benefits to families who often have great anxiety as they wait for diagnostic confirmation. 

Many studies both across Australia and internationally are in various stages of exploring the ethical, legal and societal implications (ELSI) of genomic newborn screening (gNBS) [13,14,15,16,17]. There are many issues to consider when looking to introduce population-wide screening programs such as gNBS. Engagement with parents, the general public and key stakeholders is critical to ensure a gNBS program is designed to complement existing NBS programs [14].

The TRAIL study includes a large ELSI component which aims to explore parental knowledge and understanding of gNBS, as well as the educational needs of midwives and nurses who currently offer existing NBS. Using a combination of surveys, focus groups and in-depth interviews, the TRAIL study aims to add meaningful results to the ongoing ELSI discussions happening nationally and internationally.

## 5. TRAIL SNP Methods and the Question of Big Data

There are methods, such as mass spectrometry, that enable doctors to perform low-cost, reliable SNP assays, and TRAIL will pilot these methods [18]. However, to take full advantage of having genomic data available with quick interrogation and action, we would ideally have point-of-care testing (POCT) systems to perform rapid SNP that is readily available. Whilst POCT genetic tests have been developed for other purposes, such as pharmacogenomic testing [19], to our knowledge, there are currently no POCT systems available for low-cost SNP analysis, but as the potential demand grows, this could quickly change. Having POCT instruments available in major hospitals and clinics would allow them to be linked to online systems accessing and analysing genomic NBS data when a critical patient presents with a suspected genetic disorder. This would greatly speed up the diagnosis and confidence to start treatment of a genetic disorder rather than having to start with sequencing when the patient presents to hospital.

Another possibility is that family could opt to have their NBS genomic screening data confirmed in a diagnostic setting at a time of their choosing. Thus, if their child requires urgent genomic analysis, their data could already have been moved from screening grade to diagnostic grade. The family may choose to do this at a time such as their newborn’s 6-week vaccination visit and also by using a saliva-based specimen.

There may be data quality issues associated with genomic data generated from an NBS card. These limitations may include insufficient read depth and inaccurate sequencing alignment in complex repeat patterns within the genome. These limitations of WGS may require repeat genomic testing; however, if the original NBS data and the region of interest have good-quality metrics, the data could be considered equivalent to a diagnostic-grade specimen, and therefore, no repeat testing may be required.

## 6. Conclusions

Currently, NBS relies on phenotypic data such as metabolite levels and often second-tier protocols to improve screening accuracy and reduce false positives. With new therapies for genetic diseases emerging, it is a greater challenge to find appropriate biomarkers to readily detect and diagnose these disorders, or equally, these metabolites may not show abnormal levels at the time of collection for NBS. The storage of WGS data from NBS may provide a basis of ‘data for life’, providing opportunities for future re-analysis and a closer reflection of the germline DNA. Evidence to inform this approach is currently being gathered through the previously mentioned current research study TRAIL https://kr.schn.health.nsw.gov.au/our-research/research-initiatives/newborn-gen-seq-trail (accesse on 16 October 2024).

## Data Availability

Data sharing is not applicable to this article.
